# Defining the impact of delayed antibiotic administration using a comprehensive electronic health record screen to identify sepsis

**DOI:** 10.1186/cc11790

**Published:** 2012-11-14

**Authors:** RC Arnold, SM Hollenberg

**Affiliations:** 1 Cooper University Hospital, Camden, NJ, USA

## Background

The true effectiveness of early antibiotic administration relative to the clinical identification of sepsis in a real-world setting is unknown. Previous studies described the impact of antibiotic timing within an isolated septic shock cohort. A novel electronic health record (EHR) screening tool, Clinical Vigilance™ for Sepsis, is able to identify the presence of sepsis and correlate with meaningful patient-centered outcomes. The objective was to define the impact of delayed antibiotic administration relative to the clinical identification of sepsis using an EHR alert system.

## Methods

A retrospective analysis of a prospectively compiled registry of patient EHR records in a single-center 300-bed community hospital. A consecutive assessment of all adult patients with suspected infection over a 3-month period in 2011. A physician order for intravenous antibiotics was used as a surrogate for the clinical suspicion of systemic infection (sepsis). The test variable was application of a comprehensive automated EHR screening tool, CV Alert, to identify high-risk sepsis patients based on a multifactor alert system including labs, vital signs, and treatment team documentation. The primary outcome was all cause in-hospital mortality, and a secondary outcome was hospital length of stay (LOS). Antibiotic delivery was defined *a priori *as the time a physician order was placed for intravenous antibiotics and outcomes were assessed every 12 hours prior to and subsequent to the CV Alert.

## Results

We identified 2,255 consecutive patients with suspected infection over a 3-month period from a total of 23,717 screened (9.5%). CV Alert was triggered in 867 of 2,255 (38%). Patients identified by CV Alert (*n *= 867) had an increased mortality rate (5.3% vs. 0.6%, *P *< 0.001) and increased hospital LOS (5 vs. 2 days, *P *< 0.001) compared with patients not triggering an alert (*n *= 1,388). Patients given antibiotics 0 to 12 hours after the alert had a significantly increased mortality rate (8.9% vs. 3.3%, *P *< 0.002) and longer LOS (6 vs. 4 days, *P *< 0.001) compared with patients given antibiotics 0 to 24 hours prior to alert.

## Conclusion

Among patients with suspected infection, those identified by the CV Alert had an increased mortality rate and hospital length of stay. Delayed antibiotics relative to the time of CV Alert were associated with a progressive increase in mortality rate and hospital LOS. An EHR-based screening tool applied to a real-time healthcare system could aid in the early identification of at-risk patients within a sepsis cohort.

